# Supplementary material for: Causal Relationship between Aging and Anorexia Nervosa: A White-Matter-Microstructure-Mediated Mendelian Randomization Analysis
Source: Biomedicines. 2024 Aug 16;12(8):1874. doi: 10.3390/biomedicines12081874 (PMC11351342; doi:10.3390/biomedicines12081874)
Supplement: Supplementary file 1 [file biomedicines-12-01874-s001.zip › Supplementary Figure/Figure S4/Singe SNP plot of MR analysis for TL to ubm-b-1842.pdf]

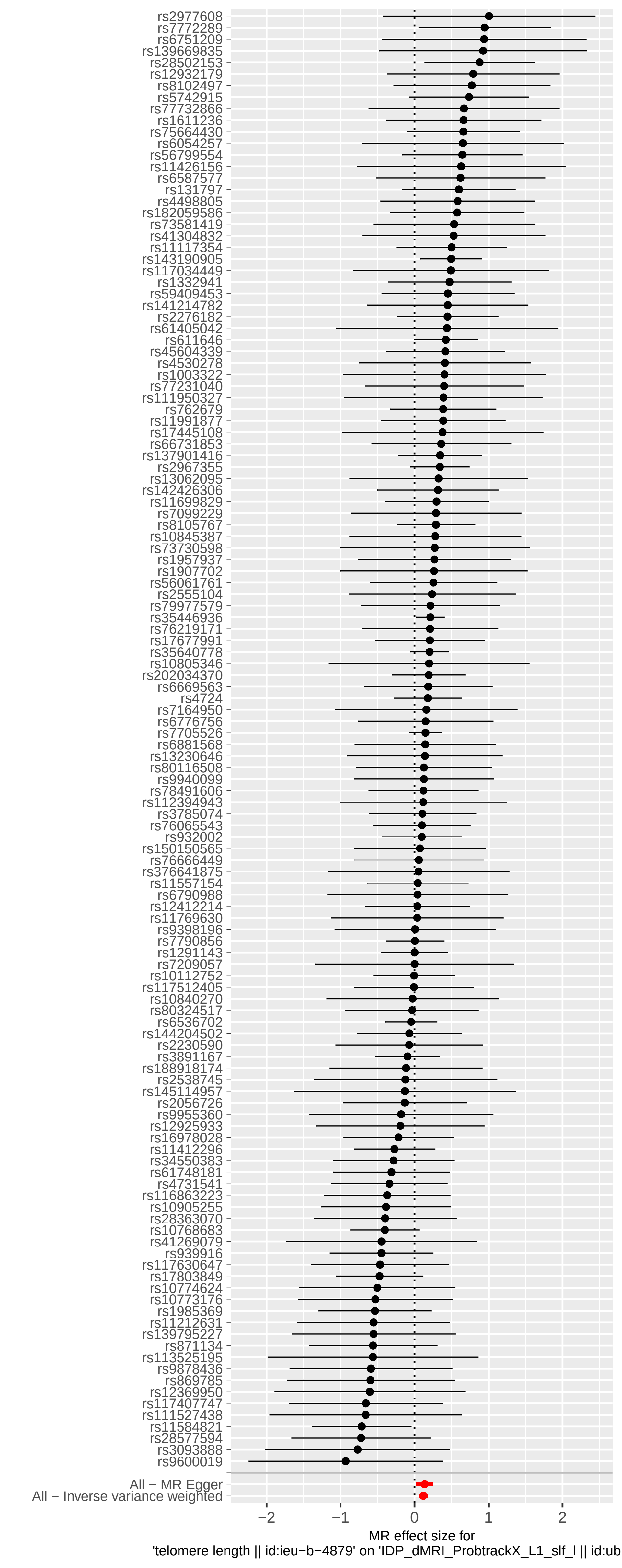
rs2977608
rs7772289
rs6751209
rs139669835
rs28502153
rs12932179
rs8102497
rs5742915
rs77732866
rs1611236
rs75664430
rs6054257
rs56799554
rs11426156
rs6587577
rs131797
rs4498805
rs182059586
rs73581419
rs41304832
rs11117354
rs143190905
rs117034449
rs1332941
rs59409453
rs141214782
rs2276182
rs61405042
rs611646
rs45604339
rs4530278
rs1003322
rs77231040
rs111950327
rs762679
rs11991877
rs17445108
rs66731853
rs137901416
rs2967355
rs13062095
rs142426306
rs11699829
rs7099229
rs8105767
rs10845387
rs73730598
rs1957937
rs1907702
rs56061761
rs2555104
rs79977579
rs35446936
rs76219171
rs17677991
rs35640778
rs10805346
rs202034370
rs6669563
rs4724
rs7164950
rs6776756
rs7705526
rs6881568
rs13230646
rs80116508
rs9940099
rs78491606
rs112394943
rs3785074
rs76065543
rs932002
rs150150565
rs76666449
rs376641875
rs11557154
rs6790988
rs12412214
rs11769630
rs9398196
rs7790856
rs1291143
rs7209057
rs10112752
rs117512405
rs10840270
rs80324517
rs6536702
rs144204502
rs2230590
rs3891167
rs188918174
rs2538745
rs145114957
rs2056726
rs9955360
rs12925933
rs16978028
rs11412296
rs34550383
rs61748181
rs4731541
rs116863223
rs10905255
rs28363070
rs10768683
rs41269079
rs939916
rs117630647
rs17803849
rs10774624
rs10773176
rs1985369
rs11212631
rs139795227
rs871134
rs113525195
rs9878436
rs869785
rs12369950
rs117407747
rs111527438
rs11584821
rs28577594
rs3093888
rs9600019
All − MR Egger
All − Inverse variance weighted
−2
−1
0
1
2
MR effect size for
'telomere length || id:ieu−b−4879' on 'IDP_dMRI_ProbtrackX_L1_slf_l || id:ub
